# PRC2/EED-EZH2 Complex Is Up-Regulated in Breast Cancer Lymph Node Metastasis Compared to Primary Tumor and Correlates with Tumor Proliferation *In Situ*

**DOI:** 10.1371/journal.pone.0051239

**Published:** 2012-12-10

**Authors:** Hongxiang Yu, Diana L. Simons, Ilana Segall, Valeria Carcamo-Cavazos, Erich J. Schwartz, Ning Yan, Neta S. Zuckerman, Frederick M. Dirbas, Denise L. Johnson, Susan P. Holmes, Peter P. Lee

**Affiliations:** 1 Division of Hematology, Department of Medicine, Stanford University, Stanford, California, United States of America; 2 Department of Statistics, Stanford University, Stanford, California, United States of America; 3 Department of Pathology, Stanford University School of Medicine, Stanford, California, United States of America; 4 Department of Surgery, Stanford University School of Medicine, Stanford, California, United States of America; 5 Department of Cancer Immunotherapeutics and Tumor Immunology, City of Hope Cancer Center, Duarte, California, United States of America; University of Hong Kong, Hong Kong

## Abstract

**Background:**

Lymph node metastasis is a key event in the progression of breast cancer. Therefore it is important to understand the underlying mechanisms which facilitate regional lymph node metastatic progression.

**Methodology/Principal Findings:**

We performed gene expression profiling of purified tumor cells from human breast tumor and lymph node metastasis. By microarray network analysis, we found an increased expression of polycomb repression complex 2 (PRC2) core subunits *EED* and *EZH2* in lymph node metastatic tumor cells over primary tumor cells which were validated through real-time PCR. Additionally, immunohistochemical (IHC) staining and quantitative image analysis of whole tissue sections showed a significant increase of EZH2 expressing tumor cells in lymph nodes over paired primary breast tumors, which strongly correlated with tumor cell proliferation *in situ*. We further explored the mechanisms of PRC2 gene up-regulation in metastatic tumor cells and found up-regulation of *E2F* genes, *MYC* targets and down-regulation of tumor suppressor gene E-cadherin targets in lymph node metastasis through GSEA analyses. Using IHC, the expression of potential EZH2 target, E-cadherin was examined in paired primary/lymph node samples and was found to be significantly decreased in lymph node metastases over paired primary tumors.

**Conclusions/Significance:**

This study identified an over expression of the epigenetic silencing complex PRC2/EED-EZH2 in breast cancer lymph node metastasis as compared to primary tumor and its positive association with tumor cell proliferation *in situ*. Concurrently, PRC2 target protein E-cadherin was significant decreased in lymph node metastases, suggesting PRC2 promotes epithelial mesenchymal transition (EMT) in lymph node metastatic process through repression of E-cadherin. These results indicate that epigenetic regulation mediated by PRC2 proteins may provide additional advantage for the outgrowth of metastatic tumor cells in lymph nodes. This opens up epigenetic drug development possibilities for the treatment and prevention of lymph node metastasis in breast cancer.

## Introduction

Axillary lymph node metastasis is the single most important prognostic factor for patient survival [1], [2] and represents the transition from local to systemic disease in breast cancer. Despite efforts made by several groups to identify lymph node metastasis-related signatures, limited information is known about molecular mechanisms underlying this key process. Some studies showed a number of genes differentially expressed between lymph node metastasis and primary tumor. These include genes implicated in extracellular matrix adhesion and a 79-gene signature with prognostic value [3], [4]. The common caveat of these studies was that they performed gene expression profiling on whole tissues consisting of heterogeneous populations of tumor and stromal cells. Therefore, subtle though biologically relevant information may be diluted and concealed by gene signatures from non-tumor components. In an effort to solve this issue, Vecchi and his colleagues used microarray profiling in combination with tissue microarray to further validate the cellular origins of genes identified in microarray studies. With this approach, they showed four genes with epithelial origin to be down-regulated in lymph node metastasis [5]. Ellsworth and colleagues performed gene expression analyses on laser capture microdissected tumor cells and found 13 up-regulated genes and 38 down-regulated genes in lymph node metastases [6]. Their data improved current understanding of lymph node metastatic process and provided important insights on potential genetic targets for the treatment of breast cancer metastases.

In this study, we performed gene expression profiling of freshly isolated tumor cells from primary breast tumor tissues and tumor positive lymph nodes with the goal of identifying therapeutic targets for the treatment of lymph node metastasis. We undertook an integrative approach by combining gene set and network based microarray analyses with validations through real-time PCR and quantitative image analysis of whole tissue sections using histological methods. We found increased expression of polycomb repression complex 2 (PRC2) genes in lymph node metastatic tumor cells compared to primary tumor cells. Additionally, over expression of EZH2, the catalytic unit of PRC2, in lymph node metastasis strongly correlated with tumor cell proliferation *in situ*. The PRC2 complex belongs to polycomb-group (PcG) proteins and functions as epigenetic repressors which silence specific sets of genes through its histone 3 lysine 27 (H3K27) methyltransferase activity [7]. This is the first demonstration of PRC2 over expression in breast cancer lymph node metastases relative to primary tumor, and opens new avenues for cancer therapeutics to target epigenetic processes for the reversal of epigenetically regulated metastatic mechanisms.

## Results

### Network Analysis Revealed Up-regulation of PRC2 Genes in Lymph Node Metastasis

**Figure 1:**
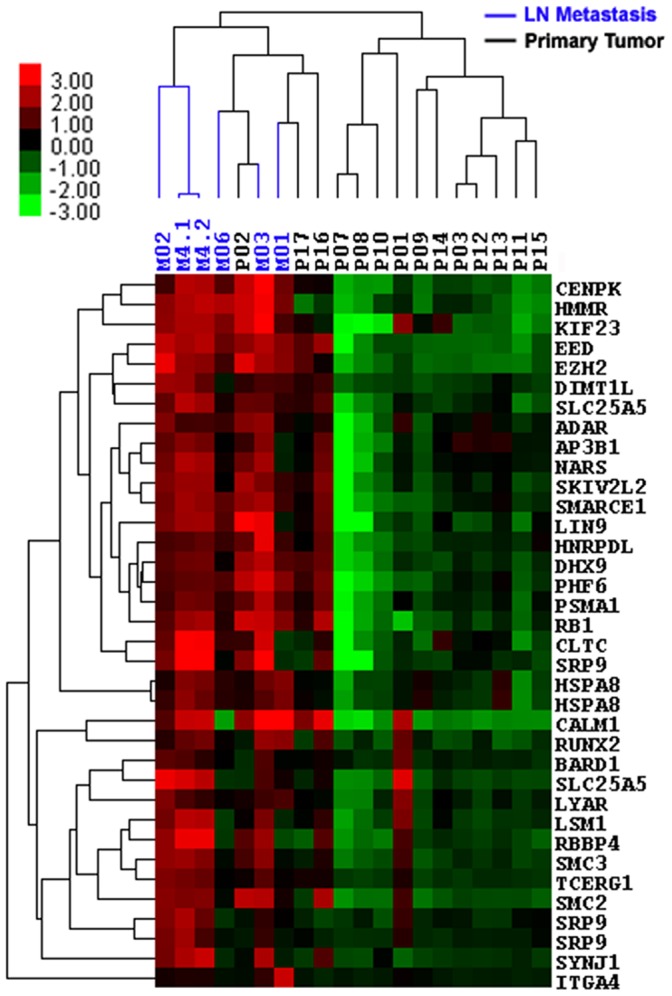
Heat Map of LN Met Set. Red and green represent up-regulation and down-regulation, respectively. Lymph node metastatic samples are indicated by blue lines and primary tumors indicated by black lines.

To capture subtle yet biologically important changes, the Gene eXpression Network Analysis (GXNA) program [8] was used to determine significantly changed networks/subnetworks between lymph node metastasis and primary tumor. This produced a network of thirty-one genes with a significant score of 15 (Table 1). This network was significantly up-regulated in lymph node metastasis compared to primary tumor (adjusted p = 0.02) and will be referred to as LN Met Set herein. The heat map representing the hierarchical clustering of these genes is shown in Figure 1. Metastatic tumor samples purified from lymph nodes (M01–M06) clustered together and showed relatively consistent over expression of LN Met Set genes compared to primary tumors. Of note, there were three paired primary tumor and lymph node metastatic samples (01–03). These metastatic tumor cells clustered with other metastatic samples rather than their matched primary tumors, indicating that the LN Met Set was able to distinguish primary tumor cells from lymph node metastatic cells.

**Table 1:** Genes up-regulated in lymph node metastasis compared to primary tumor.

Gene Symbol	Official Full Name
**Chromatin remodeling/Histone modification**	
EED	embryonic ectoderm development
EZH2	enhancer of zeste homolog 2 (Drosophila)
SMARCE1	SWI/SNF related, matrix associated, actin dependent regulator of chromatin, subfamily e, member 1
PHF6	PHD finger protein 6
RBBP4	retinoblastoma binding protein 4
**RNA processing**	
HNRPDL	heterogeneous nuclear ribonucleoprotein U (scaffold attachment factor A)
SKIV2L2	superkiller viralicidic activity 2-like 2 (S. cerevisiae)
DHX9	DEAH (Asp-Glu-Ala-His) box polypeptide 9
LSM1	LSM1 homolog, U6 small nuclear RNA associated (S. cerevisiae)
ADAR	adenosine deaminase, RNA-specific
DIMT1L	DIM1 dimethyladenosine transferase 1-like (S. cerevisiae)
LYAR	Ly1 antibody reactive homolog (mouse)
**RNA Pol II transcriptional factor**	
TCERG1	transcription elongation regulator 1
**Cell motility and Protein translocation**	
HMMR	hyaluronan-mediated motility receptor (RHAMM)
SRP9	signal recognition particle 9 kDa
**Cell division**	
CENPK	centromere protein K
KIF23	kinesin family member 23
LIN9	lin-9 homolog (C. elegans)
SMC2	structural maintenance of chromosomes 2
SMC3	structural maintenance of chromosomes 3
BARD1	BRCA1 associated RING domain 1
**Endocytosis and Transport**	
CLTC	clathrin, heavy chain (Hc)
SLC25A5	solute carrier family 25 (mitochondrial carrier; adenine nucleotide translocator), member 5
SYNJ1	synaptojanin 1
AP3B1	adaptor-related protein complex 3, beta 1 subunit
**Others**	
NARS	asparaginyl-tRNA synthetase
PSMA1	proteasome (prosome, macropain) subunit, alpha type, 1
CALM1	calmodulin 1 (phosphorylase kinase, delta)
RUNX2	runt-related transcription factor 2
HSPA8	heat shock 70 kDa protein 8
ITGA4	integrin, alpha 4

To validate our findings, we examined whether the LN Met Set identified in this study could distinguish metastasis from primary tumor in previously published breast cancer microarray datasets using Gene Set Enrichment Analysis (GSEA) [9]. We utilized breast cancer microarray dataset GSE2741, which contains microarray data of lymph node metastasis, primary tumor and normal breast tissue [10], [11]. Nonspecific filtering was performed on this breast cancer microarray dataset and 9,908 gene sets were used in the analysis. Twenty-eight genes from the LN Met Set were available in the filtered breast cancer dataset GSE2741 and used to determine whether the LN Met Set was associated with lymph node metastasis compared to primary tumor or normal breast tissue. In agreement with our data, we found a significant enrichment of the LN Met Set in lymph node metastasis compared to primary tumor or normal breast tissue using GSEA (Figure S1).

**Figure 2:**
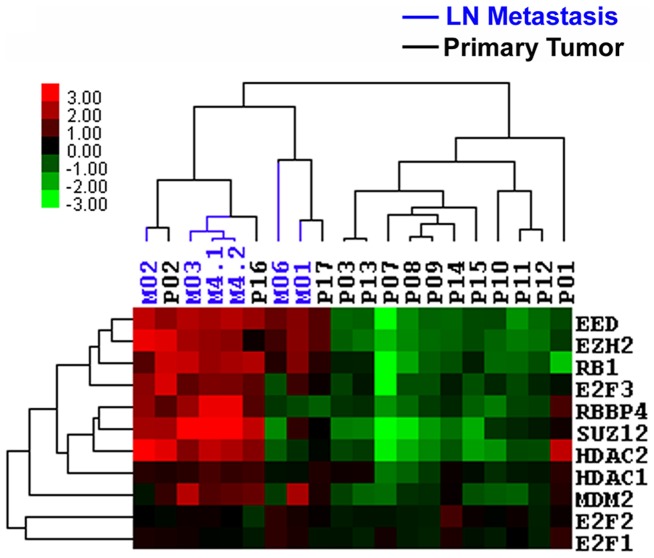
Heat map view of the PRC2 network gene expressions in primary tumor and lymph node metastasis.

To characterize the LN Met Set, we generated a protein-protein interaction network using the STRING database (http://string-db.org/) [12]. The PRC2 genes *EZH2, EED, SUZ12, RBBP4* and their interacting neighbors (*HDAC1, HDAC2, E2F1-3, MDM2*) formed a prominent tightly connected protein network. The relative expressions of these genes are presented as a heat map in Figure 2. In this network, 3 genes (*EZH2, EED, RBBP4*) were found to be significantly up-regulated in lymph node metastasis using the program GXNA. Although not identified through GXNA analyses, *E2F3* was up-regulated in 5 out of 6 lymph node metastasis and clustered with *EZH2 and EED*. However, expression of *E2F1* and *E2F2* were relatively low in all samples, possibly due to insensitive probes on the microarrays. Two additional PRC2 genes, *SUZ12* and *RBBP4*, were up-regulated in 4 out of 6 metastatic lymph node samples and co-expressed with *HDAC1-2*. Overall, the network analysis of LN Met Set highlighted up-regulations of the PRC2 complex and the pRB-E2F growth control pathway in lymph node metastasis.

### Evaluation of Elevated PRC2 Expression in Lymph Node Metastasis

**Figure 3:**
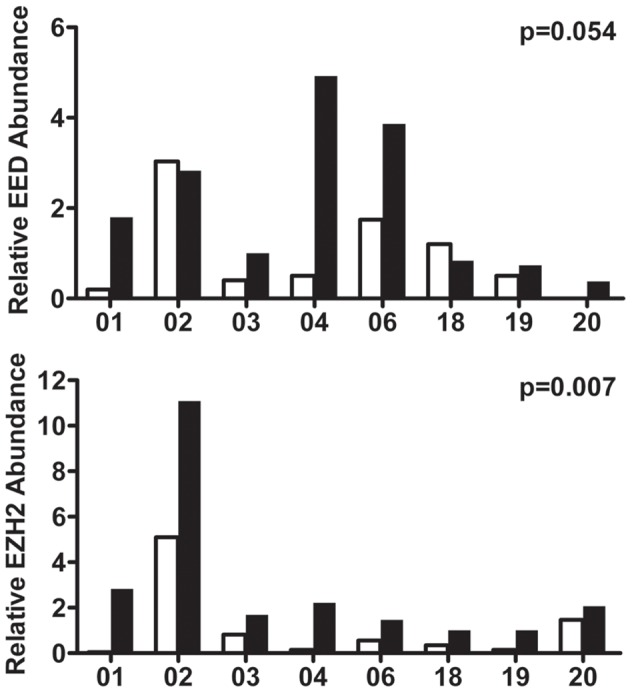
Validation of *EZH2* and *EED* mRNA expressions by real-time PCR. White bars indicate primary tumor and the adjacent black bars indicate their matched lymph node metastasis.

We validated the expression of *EZH2* and its binding partner *EED* in purified tumor cells from 8 paired primary tumor and lymph node samples (Figure 3) with real-time PCR. Paired-Wilcoxon signed rank test showed a significant increase of *EZH2* in metastatic tumor cells compared to primary tumor cells (p = 0.007); *EED* demonstrated an increased trend in 6 out of 8 paired samples (p = 0.054). Real-time PCR analyses of *RBBP4* was performed in 6 paired primary tumor and lymph node samples (Figure S2) which showed no significant difference.

**Figure 4:**
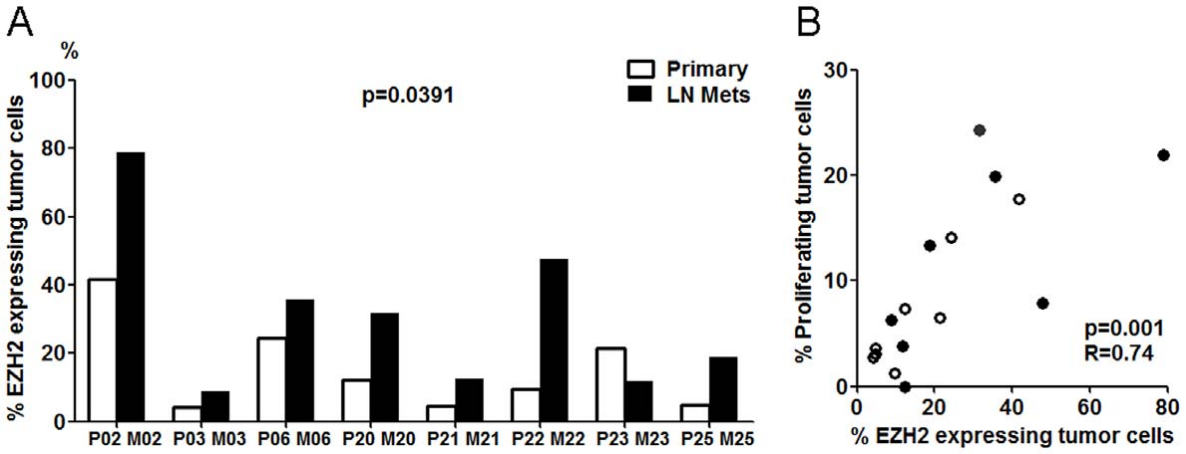
Validation of EZH2 protein expression by quantitative image analysis of whole tissue sections. A: Proportion of EZH2 expressing tumor cells in paired lymph node and primary tumor sections are presented as adjacent bars. Percentage of EZH2+ tumor cells among the total number of tumor cells are indicated by Y axis. B: Correlation between percentage of EZH2 expressing tumor cells and percentage of proliferating cells. Lymph node metastases are indicated by solid circle and primary tumors by open circle. The Pearson correlation coefficient is 0.74 with p-value 0.001.

To assess whether EZH2 was elevated at the protein level in lymph node metastasis, we determined the percentage of EZH2 expressing tumor cells in 8 paired primary breast tumor and lymph node tissue sections using immunohistochemical methods. Given that EZH2 has been associated with tumor cell proliferation [13], the proliferation marker Ki67 was included in the staining to investigate the correlation of EZH2 with tumor cell proliferation. The proportion of each phenotype in the whole tissue image was quantitatively assessed as described in methods. The proportion of EZH2 expressing tumor cells in lymph nodes was significantly higher compared to matched primary tumor cells (Figure 4A, p = 0.039). All proliferating cells expressed EZH2, although EZH2 did not always co-stain with Ki67. Pearson correlation showed a significantly positive correlation between the percentage of EZH2 expressing cells and that of proliferating cells (Figure 4B, p = 0.001, R = 0.74), demonstrating a strong association of EZH2 with tumor proliferation in both metastatic lymph nodes and primary tumors *in situ*.

### Mechanisms of PRC2 Up-regulation in Lymph Node Metastasis

To further explore the mechanisms of PRC2 gene up-regulation in lymph node metastasis, GSEA was used to investigate enrichment patterns of prior defined gene sets in lymph node metastasis and primary tumor. We used gene set C2 from GSEA Molecular Signatures Database version 3.0 (http://www.broadinstitute.org/gsea/index.jsp). The C2 collection (canonical pathways and chemical and genetic perturbations) includes 3272 gene sets collected from various sources such as online pathway databases, publications in PubMed, and knowledge of domain experts.

GSEA analysis of the gene sets C2 showed that 18 gene sets were up-regulated in lymph node metastasis and 19 gene sets were up-regulated in primary tumor (Table S1). Compared to primary tumor, lymph node metastasis was enriched with gene sets associated with cell proliferation, aggressive solid tumor phenotypes, metastatic processes, undifferentiated tumor grade and poor survival. Genes regulated by several known key regulators of PRC2 components were up-regulated in lymph node metastasis, including *Myc* targets and *E2F* genes. Of particular interest, genes activated by the tumor suppressor gene E-cadherin (*CDH1*) were significantly down-regulated in lymph node metastasis, although *CDH1* itself did not show differences at the transcriptional level. Lymph node metastasis also over expressed genes associated with resistance to doxorubicin, a cancer chemotherapy drug. Concurrently, lymph node metastasis down-regulated gene sets associated with drug-responses, including cisplatin, a cancer chemotherapy drug and MP470, a novel c-Kit/AXL kinase inhibitor. Collectively, the transcriptional scheme of lymph node metastatic cells exhibited a highly proliferative and aggressive phenotype.

**Figure 5:**
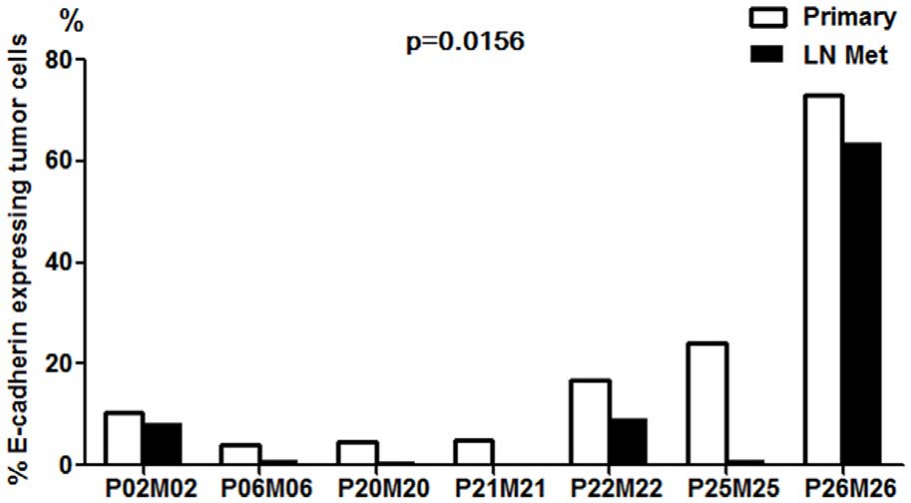
Immunohistochemical staining and quantitative image analysis of whole tissue sections of E-cadherin protein expression. Proportion of E-cadherin expressing tumor cells in paired lymph node and primary tumor sections are presented as adjacent bars. Percentages of E-cadherin+ tumor cells among the total number of tumor cells are indicated by Y axis.

Previously it has been shown that EZH2 mediates transcriptional silencing of E-cadherin by trimethylation of H3K27 [14]. To determine whether E-cadherin was repressed at the protein level in lymph node metastasis, we assessed the percentage of E-cadherin expressing tumor cells in 7 paired primary tumor and lymph node tissue sections using immunohistochemical methods. The proportion of E-cadherin expressing tumor cells in the whole tissue image was quantitatively assessed as described in methods. The proportion of E-cadherin expressing tumor cells in lymph nodes was significantly lower compared to matched primary tumor cells (Figure 5, p = 0.031).

## Discussion

Metastatic tumor cells arise within the primary tumor cell population and acquire additional genetic and epigenetic changes to enable metastatic outgrowth to the draining lymph nodes and distant organs. Microarray analysis has been widely used to study genetic changes implicated in tumor initiation, progression and metastasis [3]–[5], [10], [11]. The genes list identified by our study is largely not overlapping with previous findings. This discrepancy is not uncommon for gene expression studies due to different study design, microarray platforms, analytical approaches and different sample sizes of different cohorts. Previous studies have provided important insights on genetic abnormalities in the lymph node metastatic process. However, epigenetic alterations in cancer development, such as promoter methylations and histone modifications are less well studied. Indeed, emerging evidence point to a key role for epigenetic alterations in cancer and metastasis [14], [15]. Many tumor suppressors were found to be inactivated by epigenetic silencing through histone modifications, providing tumor cells with selective advantages for clonal expansion and growth [16]. Histone modifying enzymes alter the chromatin architecture and regulate the binding of transcription factors and hence, gene expression. An aberrant expression in these histone modifying enzymes is known to play a role in cancer progression [7], [17].

By profiling purified tumor cells from human breast tumor and lymph nodes, we discovered an up-regulation of epigenetic silencing complex PRC2 core subunits (EED-EZH2) in lymph node metastatic tumor cells compared to primary tumor cells. The PRC2 complex functions as epigenetic repressors by silencing specific sets of genes through its histone 3 lysine 27 (H3K27) methyltransferase activity [7]. Molecular mechanisms involving PRC2 proteins have attracted significant attention in cancer research due to their critical role in controlling differentiation and tumorigenesis [7], [18], [19]. The PRC2 complex is composed of three core subunits*: EZH2*, *EED* and *SUZ12*. *EZH2* is the catalytically active unit of the PRC2 complex which participates in the transcriptional repression of target genes by trimethylation of H3-K27, providing an epigenetic mark for the PRC1 complex binding [7]. The polycomb protein EZH2 is emerging to be a key driver for tumor formation and has been implicated in prostate cancer metastasis [20], [21]. Additionally, EZH2 has been found to be over expressed in both primary and metastatic breast cancer as compared to normal tissue [22]. *In vitro* studies suggested that EZH2 drives neoplastic transformation of breast epithelial cells by promoting anchorage-independent and invasive growth [22]. Our findings demonstrated a further up-regulation of EZH2 in lymph node metastasis relative to primary tumor, and its positive association with tumor proliferation, suggesting a pivotal role of EZH2 in the lymph node metastatic process. Previous studies have revealed a number of possible mechanisms of EZH2 up-regulation in various cancers [23]–[30]. We found concurrent up-regulation of several known key regulators of PRC2 genes in lymph node metastasis, including *MYC* and *E2F* through microarray analyses. Bracken et al. previously showed that expressions of the two PRC2 core subunits EZH2 and EED were tightly controlled by the E2F transcription factors. EZH2 and EED are required for cell growth to confer a proliferative advantage and act as essential downstream mediators of E2F function [30]. In addition to a significant up-regulation of PRC2/*EED-EZH2* genes in LN metastasis, our microarray data showed an increase of *E2F3* in 5 out of 6 lymph node metastasis compared to primary tumor. As such, PRC2/EED-EZH2 components could act downstream of *E2F* genes to provide additional growth advantage for LN metastatic tumor cells. Additionally, MYC may also be responsible for the up-regulation of the PRC2 genes through regulation of microRNA expressions in lymph node metastases. MYC not only stimulates EZH2 expression by repression of its negative regulator miR-26a [25], but also modulates E2F1 through miR-17-5p and miR-20a [26]. The microRNA profiles in breast cancer lymph node metastases are largely unknown. Therefore further investigations are warranted to clarify whether MYC plays a major role in regulating EZH2 over expression in LN metastasis.

Furthermore, we found that lymph node metastatic tumor cells showed down-regulation of genes activated by tumor suppressor gene E-cadherin through GSEA enrichment analyses. Further validation of E-cadherin expression through IHC revealed a significant decrease in E-cadherin expressing tumor cells in LN metastasis compared to paired primary tumor, which coincided with an up-regulation of EZH2 in LN metastasis. E-cadherin down-regulation is one of the hallmarks of epithelial-mesenchymal transition (EMT), which is essential for tumor invasion and metastasis [31], [32]. Previously, it has been shown that EZH2 mediates transcriptional silencing of E-cadherin by trimethylation of H3K27 and that HDAC inhibitors could attenuate tumor invasion by blocking EZH2- mediated repression of E-cadherin [14]. Moreover, E-cadherin was found to be the most frequently methylated gene in breast cancer sentinel lymph node metastasis and the hypermethylation of E-cadherin was frequently observed in lymph node metastasis (90%) more than in primary tumors (48%) [33]. Deregulation of PRC2 proteins in tumor cells may not only lead to silencing of tumor suppressors which promote tumorigenesis, but could also involve promoting EMT genes such as E-cadherin to facilitate tumor invasion. Given the connection between EZH2 and E-cadherin in the process of breast cancer lymph node metastasis, EZH2 could be an appealing therapeutic target for the treatment of breast cancer metastasis.

Previous microarray studies have focused on potential genetic targets for treating breast cancer metastasis. Our findings showed an over expression of epigenetic silencing complex PRC2/EED-EZH2 in breast cancer lymph node metastasis compared to primary tumor, suggesting epigenetic regulations mediated by PRC2 proteins may provide additional advantage for the outgrowth of lymph node metastasis. In the lymph node metastatic process, PRC2/EED-EZH2 might act downstream of E2F genes and promote EMT through repression of E-cadherin. Epigenetic regulation represents a key, yet poorly understood mechanism contributing to the metastatic process. Future studies are warranted to identify critical epigenetic regulatory mechanisms involved in the metastatic process. These findings provide novel insights into potential therapeutic strategies using epigenetic agents for the treatment/prevention of metastatic disease.

## Materials and Methods

### Ethics Statement

Written informed consent was obtained from all patients, and the study was approved by Stanford University’s Institutional Review Board.

### Clinical Samples

A total of 20 primary breast tumors and 9 metastatic lymph nodes (LNs) were obtained from breast cancer patients at Stanford Hospital. The patient and tumor characteristics are shown in Table S2. Microarray analysis was performed on 14 primary breast tumors and 6 metastatic lymph node samples, of which there were 3 paired tissues. These 3 paired tissues and an additional 10 paired tissues were used for real-time PCR validations.

### Isolation of Tumor Cells Using Flow Cytometry

Fresh breast tumors and tumor positive lymph nodes were minced and enzymatically dissociated with 200 units/ml type III Collagenase (Worthington Biochemical Corp.) and 10 Kunitz units/ml DNase I (Sigma) for 30 minutes to 1 hour at 37°C. The digestion process was stopped by addition of M199 media containing 10% FBS. Single cell suspensions were generated by filtering cells through a 70 micron cell strainer followed by a 40 micron cell strainer (BD Biosciences). Cells were stained with pan-leukocyte marker CD45 PE-Cy7, fibroblast marker CD140β PE (both BD Biosciences), epithelial specific antigen ESA FITC (Biolegend), and a dead cell exclusion marker ViViD (Invitrogen). ESA^+^CD45^−^CD140β^−^ cells were sorted using FACSAria (BD Bioscience) with gating strategies shown in Figure S3. Purified cells were homogenized in 1 mL TRIzol (Invitrogen) and stored at −80°C. The presence of tumor cells in the specimens were confirmed by H&E staining of a portion of the received tissues and examined by pathologists.

### Whole Genome Microarray and Data Analysis

Total RNA was extracted using the TRIzoL method and amplified using TrueLabeling-PicoAMP™ kit (QIAGEN), followed by Cy3/Cy5 labeling (GE healthcare). Cy-labeled patient samples were mixed with the same amount of reverse color Cy-labeled universal human reference (UHR) cRNA (Stratagene Corp.) and hybridized to Agilent's Whole Human Genome Microarray 4×44 K. Image files were generated from microarray slides using Agilent Microarray Scanner G2505B. Quantile normalization and LOESS normalization were used to normalize the raw data. All arrays were calibrated to the same scale and variance stabilizing transformation was applied to the data. After normalization, the generalized log ratio values for each gene were taken to obtain a relative expression level in samples over the UHR. Array data are MIAME compliant and available at National Center for Biotechnology Information’s Gene Expression Omnibus (GSE30480). Additional nonspecific filtering was performed using R-package and 8386 genes were used for the analyses. Briefly, multiple probes for the same genes were averaged when they were highly correlated; otherwise they were left as is. Probes without entrez ID or Gene Ontology annotation were removed. Variance filtering was used to remove genes which showed little or no variability. Average linkage was performed using Cluster 3.0 and heat maps were generated using JavaTreeview. The STRING database was used to generate protein-protein networks [12].

### Gene eXpression Network Analysis (GXNA)

GXNA was employed for finding significantly changed networks/subnetworks [8]. This program is designed to find the most perturbed subnetwork in a gene interaction network, where the genes are scored according to how different they are between the two groups. The first step involved finding significant scores for a relevant subset of genes. This score was generated using two-sample t-test comparing the two groups: metastatic tumor cells and primary tumor cells. Subsequently, the score data was combined with the most up to date protein interaction network [34] to generate a skeleton network. The GXNA program was used to search this ‘skeleton network’ with simulated annealing to deliver the most significantly changed subnetworks. The statistical significance was determined by the weighted sum of the absolute values of the t-statistics and multiple testing corrections were applied using randomized permutation and bootstrap procedures.

### Gene Set Enrichment Analysis (GSEA)

GSEA was used to determine whether the members of a given gene set were randomly distributed throughout the differentially expressed genes between two phenotypes or associated with a particular phenotype [9]. The t-test was used as the metrics for ranking genes and gene set was chosen as the permutation type since the sample size was less than 7 in this study. The number of permutations was set to 10,000 and gene sets with less than 10 genes or more than 500 genes were excluded from the analysis. A normalized enrichment score (NES) was calculated for each gene set to represent the degree in which it was enriched in one phenotype. The nominal p-value, the false discovery rate (FDR), family wise-error rate (FWER) corresponding to each NES were calculated. A NES with a nominal p-value <0.05, FDR <0.05, FWER <0.05 were considered statistically significant.

### Real-time PCR Analysis

Total RNA was reverse transcribed using sensiscript reverse transcriptase (QIAGEN). Real-time PCR reactions were performed in duplicates with iQ™ SYBR® Green Supermix (Bio-Rad Laboratories) and specific PCR primers (Table S3) using the iCycler iQTM (Bio-Rad Laboratories). The expression of each gene was normalized to GAPDH and analyzed using the comparative C_T_ method. The relative expression of the target gene was expressed as a fold change over UHR. Two-sided paired-Wilcoxon signed rank test was used to assess the statistical significance of differential gene expressions. P-values <0.05 were considered statistically significant.

### Whole Tissue Section Imaging and Quantitative Image Analysis

Immunohistochemistry was performed on formalin fixed paraffin-embedded tissue sections using a biotin-free polymer detection system. Prior to staining, tissue sections were deparaffinized and antigens retrieved with Diva Decloaker™ in the Decloaking Chamber. A three step staining procedure was performed on tissue sections for the detection of 1) EZH2, proliferation marker Ki67 and pan cytokeratin marker AE1/AE3 and a two step staining procedure was used for the detection of 2) E-cadherin and pan cytokeratin marker AE1/AE3. For the former, tissue sections were incubated with rabbit monoclonal antibody Ki67, followed by incubations with anti-rabbit ALP secondary antibody and color developed by chromogen Vulcan Fast Red. Endogenous peroxidase was blocked by incubation in PEROXIDAZED 1 for 10 minutes and mouse monoclonal antibody EZH2 was applied to the tissue sections followed by anti-mouse HRP secondary antibody. The color for EZH2 was developed by incubating with chromogen DAB. A denaturing solution was applied to denature bound antibodies before staining with the pan cytokeratin antibody (mouse monoclonal, clone AE1/AE3), followed by anti-mouse ALP secondary antibody and color was developed with chromogen Ferangi Blue. For the latter, tissue sections were blocked with PEROXIDAZED 1, incubated with mouse monoclonal primary antibody E-cadherin followed by anti-mouse ALP and color developed by Vulcan Fast Red. A denaturing solution was applied before staining with AE1/AE3, followed by anti-mouse ALP secondary antibody and color was developed with chromogen Ferangi Blue. All tissue sections were counterstained in CAT Haematoxylin and mounted. All antibodies and reagents were purchased from Biocare Medical (Biocare Medical).

Whole tissue section images were acquired at 200×magnification using the imaging system Vectra™ [8] as previously described [35]. The multi-chromogen stained image was decomposed into individual stains according to their spectrums. Pseudo-colors were assigned for enhanced visualization (Figure S4). Images were analyzed with GemIdent to enumerate 1) EZH2/Ki67 double positive tumor cells, EZH2 or Ki67 positive tumor cells, EZH2/Ki67 negative tumor cells and E-cadherin positive or negative tumor cells and 2) E-cadherin/AE1-AE3 double positive tumor cells and tumor cells only. The percentage of each phenotype was expressed by dividing the cell number of the phenotype with the total number of tumor cells. Two-sided paired-Wilcoxon signed rank test was used to assess the statistical significance of the percentages of each phenotype. Pearson correlation was used to evaluate the correlation of EZH2 expression and tumor cell proliferation. P-values <0.05 were considered significant.

## Supporting Information

Figure S1 **GSEA enrichment results of LN Met Set in breast cancer microarray dataset GSE2741.** A: Lymph node metastasis vs Primary Tumor. B: Lymph node metastasis vs Normal.
(TIF)

Figure S2 **Validation of RBBP4 mRNA expressions by real-time PCR.** White bars indicate primary tumor and the adjacent black bars indicate their matched lymph node metastasis.
(TIF)

Figure S3 **FACS plots indicating sorting gates for isolation of tumor cells (ESA+CD45-CD140β-ViVID-).** First, fibroblasts and dead cells were excluded by gating CD140β-ViVID- cells on a CD140β versus ViVID plot. Within the CD140β- live cells gate, a further gate was set on a CD45 versus ESA plot to exclude immune cells and identify ESA+ cells as indicated in the plots. Left Panel: tumor tissue; Right Panel: lymph node metastasis.
(TIF)

Figure S4 **Decomposition of multiple chromogen stained histological sections.** Vectra is able to decompose the original image into individual stains according to the corresponding spectrum for each chromogen. Pseudocolor can be assigned for each chromogen for better visualization. A: 200× original image. B: pseudo-colored image of blue membrane staining of cytokeratin, green nuclear staining of EZH2 and red nuclear staining of Ki67. C: pseudo-colored image of blue membrane staining of cytokerain and green nuclear staining of EZH2. D: pseudo-colored image of blue membrane staining of cytokerain and red nuclear staining of Ki67.
(TIF)

Table S1 Gene sets (C2) differentially expressed in lymph node metastasis vs primary tumor.
(XLS)

Table S2 Patient and Tumor Characteristics.
(XLS)

Table S3 PCR primer pairs.
(XLS)
