# Supplementary figures and images for: PRC2/EED-EZH2 Complex Is Up-Regulated in Breast Cancer Lymph Node Metastasis Compared to Primary Tumor and Correlates with Tumor Proliferation In Situ
Source: PLoS One. 2012 Dec 10;7(12):e51239. doi: 10.1371/journal.pone.0051239 (PMC3519681; doi:10.1371/journal.pone.0051239)

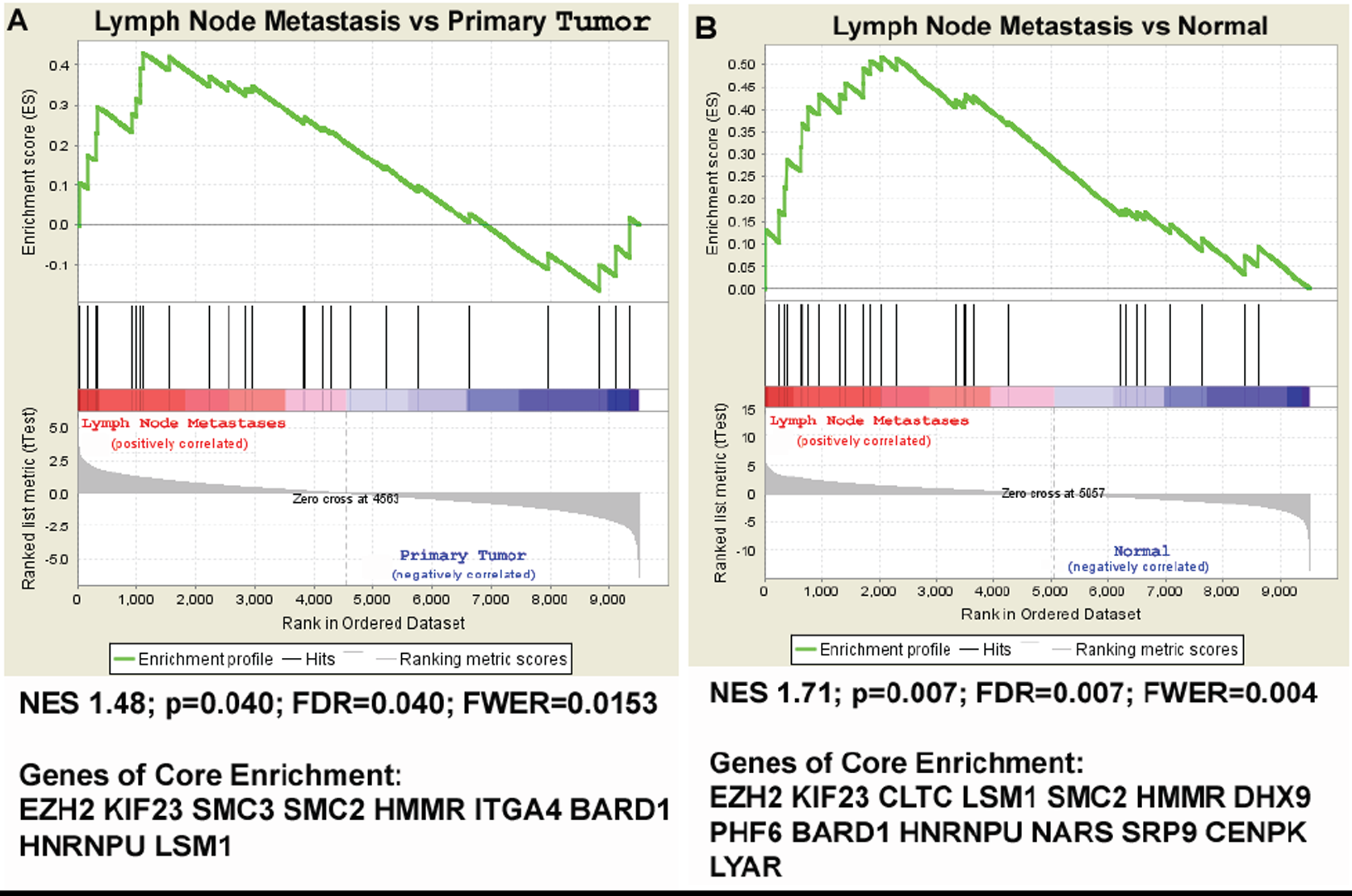

Supplement: Figure S1 — GSEA enrichment results of LN Met Set in breast cancer microarray dataset GSE2741. A: Lymph node metastasis vs Primary Tumor. B: Lymph node metastasis vs Normal. (TIF) [file pone.0051239.s001.tif]

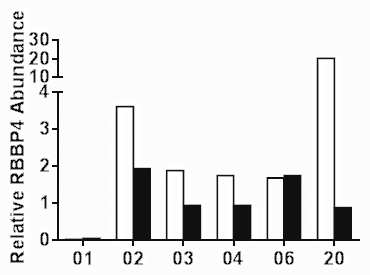

Supplement: Figure S2 — Validation of RBBP4 mRNA expressions by real-time PCR. White bars indicate primary tumor and the adjacent black bars indicate their matched lymph node metastasis. (TIF) [file pone.0051239.s002.tif]

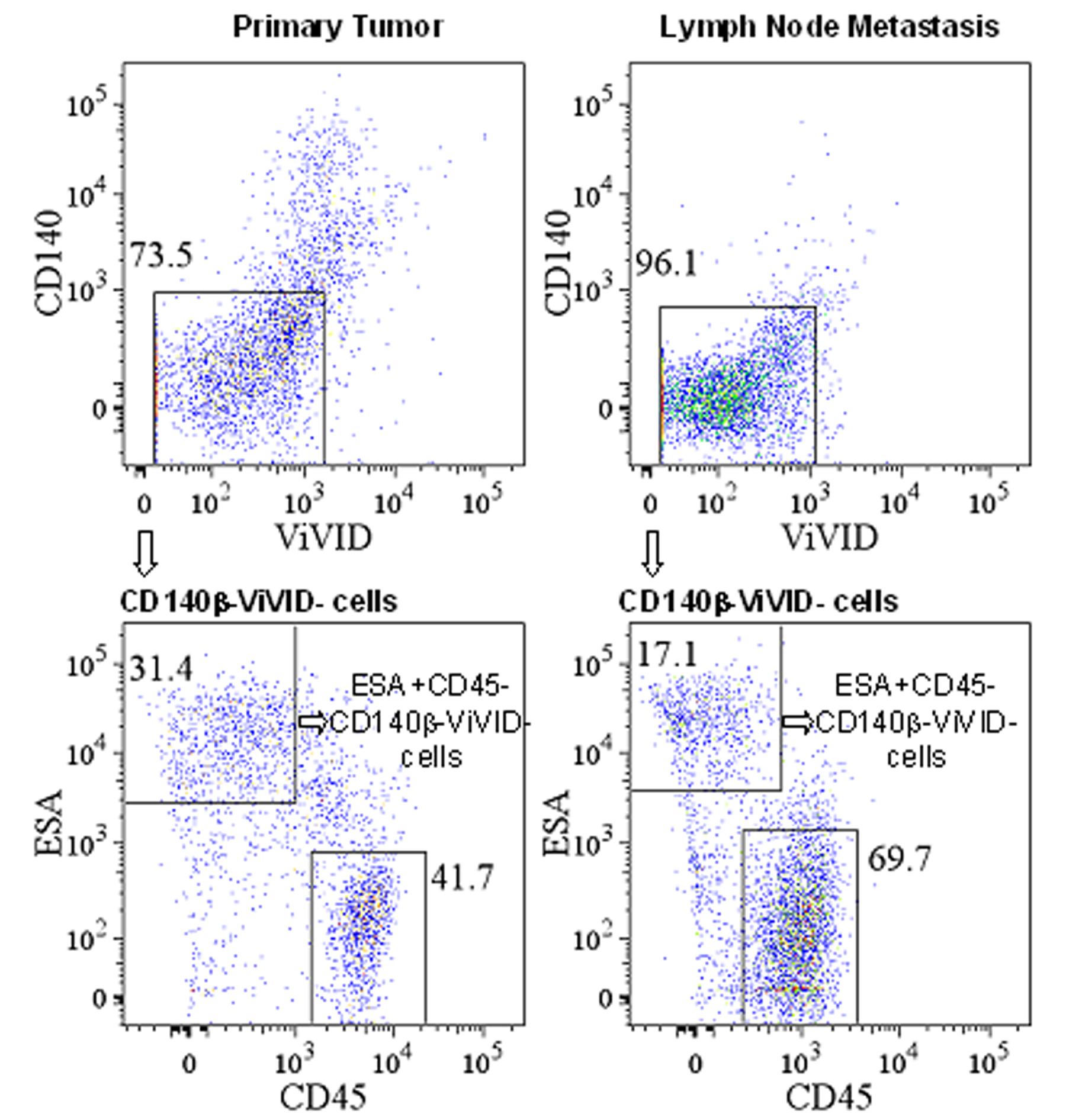

Supplement: Figure S3 — FACS plots indicating sorting gates for isolation of tumor cells (ESA+CD45-CD140β-ViVID-). First, fibroblasts and dead cells were excluded by gating CD140β-ViVID- cells on a CD140β versus ViVID plot. Within the CD140β- live cells gate, a further gate was set on a CD45 versus ESA plot to exclude immune cells and identify ESA+ cells as indicated in the plots. Left Panel: tumor tissue; Right Panel: lymph node metastasis. (TIF) [file pone.0051239.s003.tif]

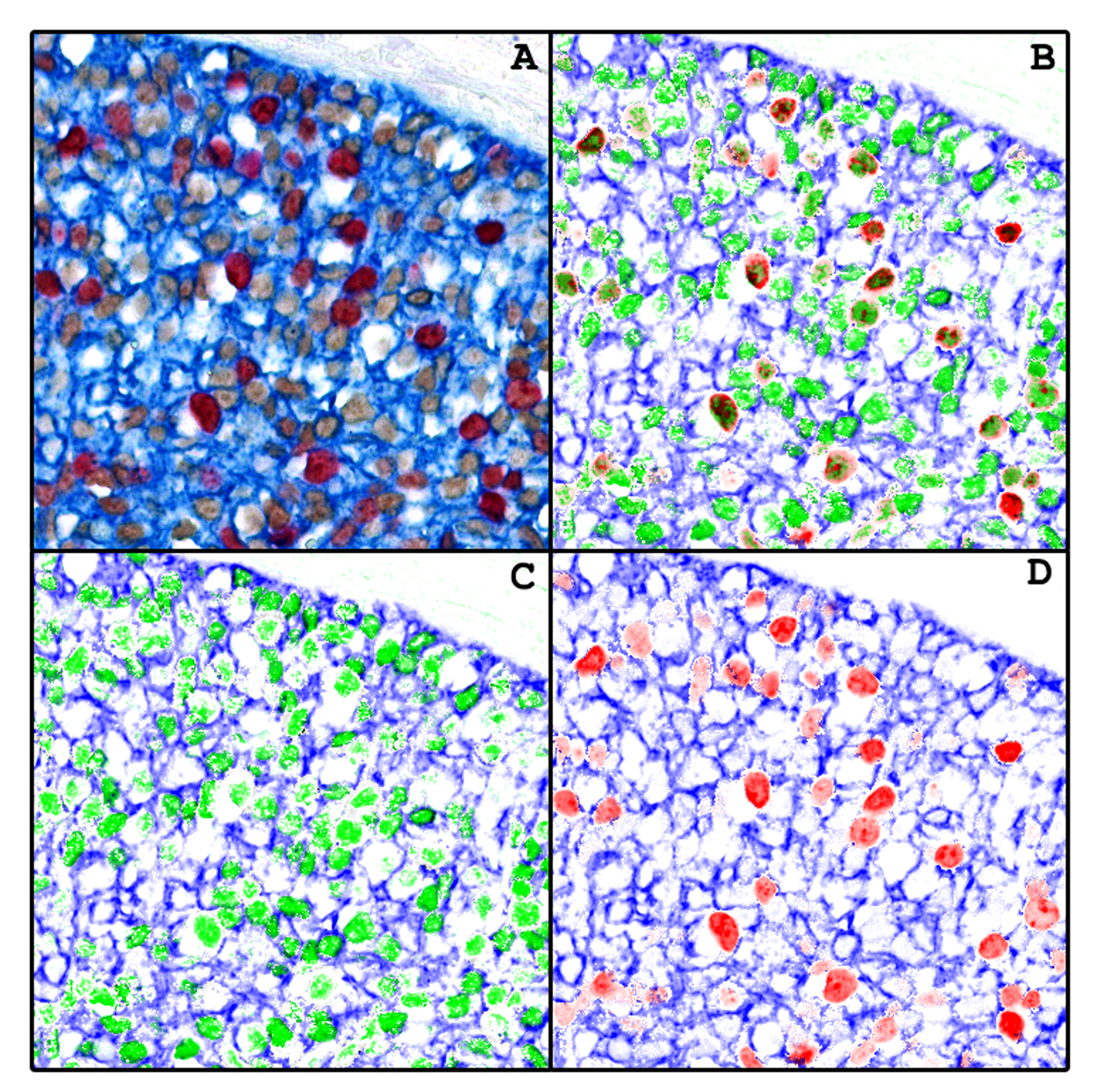

Supplement: Figure S4 — Decomposition of multiple chromogen stained histological sections. Vectra is able to decompose the original image into individual stains according to the corresponding spectrum for each chromogen. Pseudocolor can be assigned for each chromogen for better visualization. A: 200× original image. B: pseudo-colored image of blue membrane staining of cytokeratin, green nuclear staining of EZH2 and red nuclear staining of Ki67. C: pseudo-colored image of blue membrane staining of cytokerain and green nuclear staining of EZH2. D: pseudo-colored image of blue membrane staining of cytokerain and red nuclear staining of Ki67. (TIF) [file pone.0051239.s004.tif]
